# Artificial Intelligence-Guided Neuromodulation in Heart Failure with Preserved and Reduced Ejection Fraction: Mechanisms, Evidence, and Future Directions

**DOI:** 10.3390/jcdd12080314

**Published:** 2025-08-19

**Authors:** Rabiah Aslam Ansari, Sidhartha Gautam Senapati, Vibhor Ahluwalia, Gianeshwaree Alias Rachna Panjwani, Anmolpreet Kaur, Gayathri Yerrapragada, Jayavinamika Jayapradhaban Kala, Poonguzhali Elangovan, Shiva Sankari Karuppiah, Naghmeh Asadimanesh, Anjani Muthyala, Shivaram P. Arunachalam

**Affiliations:** 1 Digital Engineering & Artificial Intelligence Laboratory (DEAL), Department of Critical Care Medicine, Mayo Clinic, Jacksonville, FL 32224, USA; rabbiahaslam1996@gmail.com (R.A.A.); rachnakukreja7@gmail.com (G.A.R.P.); anmolpreetkgrewal@gmail.com (A.K.);; 2 Department of Internal Medicine, Texas Tech University Health Sciences Center, El Paso, TX 79905, USA; senapati.sidhartha36@gmail.com; 3 Department of Internal Medicine, Nazareth Hospital, Philadelphia, PA 19152, USA; 4 Department of Cardiovascular Sciences, East Carolina University, Greenville, NC 27858, USA; 5 Division of Pulmonology & Department of Critical Care Medicine, Mayo Clinic, Jacksonville, FL 32224, USA

**Keywords:** heart failure, HFpEF, HFrEF, artificial intelligence, neuromodulation, personalized medicine, vagus nerve stimulation, baroreflex activation therapy, closed-loop systems, remote health monitoring, cardiovascular technology

## Abstract

Heart failure, a significant global health burden, is divided into heart failure with reduced ejection fraction (HFrEF) and preserved ejection fraction (HFpEF), characterized by systolic dysfunction and diastolic stiffness, respectively. While HFrEF benefits from pharmacological and device-based therapies, HFpEF lacks effective treatments, with both conditions leading to high rehospitalization rates and reduced quality of life, especially in older adults with comorbidities. This review explores the role of artificial intelligence (AI) in advancing autonomic neuromodulation for heart failure management. AI enhances patient selection, optimizes stimulation strategies, and enables adaptive, closed-loop systems. In HFrEF, vagus nerve stimulation and baroreflex activation therapy improve functional status and biomarkers, while AI-driven models adjust stimulation dynamically based on physiological feedback. In HFpEF, AI aids in deep phenotyping to identify responsive subgroups for neuromodulatory interventions. Clinical tools support remote monitoring, risk assessment, and symptom detection. However, challenges like data integration, ethical oversight, and clinical adoption limit real-world application. Algorithm transparency, bias minimization, and equitable access are critical for success. Interdisciplinary collaboration and ethical innovation are essential to develop personalized, data-driven, patient-centered heart failure treatment strategies through AI-guided neuromodulation.

## 1. Introduction

### 1.1. Epidemiology: Prevalence and Incidence

Heart failure (HF) is a common condition with a 20% to 45% lifetime prevalence after 45 years of age. Prior data suggest that 0.7–0.8% of all Emergency Department (ED) visits are for HF, corresponding to nearly one million ED visits per year in the United States. This has substantial public health implications as overall costs related to HF exceed $30 billion annually, with ED and inpatient care contributing substantially to this [1]. Heart Failure (HF) is a heterogeneous, life-threatening syndrome affecting over 60 million people worldwide. It is associated with significant morbidity and mortality, diminished quality of life, and imposes a substantial burden on healthcare systems due to increased resource utilization and expenditures [2].

The 2019 Heart Failure Association (HFA) ATLAS provided comprehensive estimates of heart failure epidemiology across Europe, reporting a median prevalence of 17.2 cases per 1000 individuals, with rates ranging from ≤12 per 1000 in Greece and Spain to over 30 per 1000 in Lithuania and Germany [3]. The prevalence of heart failure in the United States was estimated at 2.4% in 2012, while prevalence rates in Asia range from 1.3% to 6.7%. Notably, the global prevalence of heart failure is projected to rise further, driven by an aging population, advances in the treatment of ischemic heart disease, and the availability of effective evidence-based therapies that prolong survival, particularly in patients with heart failure with reduced ejection fraction (HFrEF) [4].

However, data on the prevalence of specific heart failure phenotypes—stratified by ejection fraction into HFrEF, heart failure with mildly reduced ejection fraction (HFmrEF), and heart failure with preserved ejection fraction (HFpEF)—remain limited, largely due to the absence of EF assessment in many large-scale registries and administrative datasets. Most epidemiological data across the EF spectrum are derived from Western registries, where HFrEF (EF < 40%) accounts for approximately 50% of heart failure cases, while HFmrEF and HFpEF each represent about 20–25% of cases [4]. In the United States, data from the Get With The Guidelines-Heart Failure (GWTG-HF) registry indicate among hospitalized heart failure patients, 39% had HFrEF (EF < 40%), 14% had HFmrEF (EF 40–50%), and 47% had HFpEF (EF > 50%) [5].

Information on the incidence of heart failure is even scarcer than data on its prevalence. According to the HFA Atlas, the median annual incidence of heart failure in Europe was 3.2 cases per 1000 person-years, with rates varying from less than 2 per 1000 in Italy to 6 or more per 1000 in Estonia and Germany during 2018–2019 [6]. Between 2000 and 2010, the age- and sex-adjusted incidence of heart failure in the United States decreased from 3.2 to 2.2 cases per 1000 person-years [6]. Notably, in the Olmsted County cohort in the United States, 52.5% of patients with newly diagnosed heart failure had HFpEF (EF ≥ 50%). The age- and sex-adjusted incidence of heart failure declined significantly for both HFrEF and HFpEF, with an overall reduction of 37%. This decrease was more pronounced for HFrEF (−45%) compared to HFpEF (−28%), and greater among females (−43%) than males (−29%) [7].

### 1.2. Economic Burden and Clinical Challenges of HFpEF and HFrEF

A significant and increasing economic burden is imposed worldwide by heart failure (HF), which includes HFpEF and HFrEF. An estimated 6 million people in the US are thought to have HF, and by 2030, the disease is expected to cost $70 billion a year, with HFpEF or HFmrEF thought to affect roughly half of these patients [8,9]. The cost of treating heart failure in the United States is around $30,000 per patient per year, with inpatient hospitalizations making up almost half of direct costs.

Despite shorter hospital stays, the number of HF hospitalizations is still increasing [9]. According to recent modeling studies, stable HF therapy, non-cardiovascular and cardiovascular death expenses, and repeated hospitalizations are the main causes of the $123,900 total discounted cost of care over ten years for patients with symptomatic HFpEF or HFmrEF [10]. Although the economic impact varies by area worldwide, a cost-of-illness research from the Philippines showed that both HFpEF and HFrEF were responsible for large national healthcare expenditures, indicating that the burden is consistently high [11]. Both HFpEF and HFrEF are clinically linked to high rates of hospitalization and cardiovascular death; HFpEF patients have notably high readmission rates, which further increase the use of healthcare resources and associated expenses [10].

Disparities in access to evidence-based treatments and financial concerns increase the burden on patients and healthcare systems alike, while the growing frequency of HFpEF, particularly in older persons, and the absence of focused medicines provide continuous clinical challenges [12]. To overcome these obstacles, system-level initiatives to increase access, cost, and preventive care are just as important as therapeutic advancements [8,9].

### 1.3. Shared and Distinct Pathophysiologic Mechanisms

HFrEF and HFpEF are two different clinical conditions that differ in important causes but share several pathophysiological characteristics. Systemic inflammation, endothelial dysfunction, and altered myocardial energetics are major contributors to HFpEF, which is becoming better acknowledged as a syndrome of multi-morbidity, especially in patients with obesity, diabetes, hypertension, and advanced age [13,14].

HFpEF is a complex syndrome characterized by preserved ejection fraction but significant diastolic dysfunction and increased ventricular stiffness. Beyond these structural changes, recent insights underscore that systemic inflammation, endothelial dysfunction, and altered myocardial energetics are major, intertwined contributors to its pathogenesis [15]. This systemic inflammation is often closely related to underlying metabolic and vascular comorbidities, such as obesity, diabetes, and hypertension, leading to myocardial fibrosis, impaired nitric oxide bioavailability, and inefficient energy utilization, all of which contribute to the heart’s inability to relax and fill properly [15].

HFpEF is characterized by increased passive myocardial stiffness, which is caused by both intrinsic cardiomyocyte alterations, such as changes in titin isoforms and phosphorylation status, and extracellular matrix remodeling, such as increased collagen types I and III, enhanced cross-linking, and decreased collagenase activity [15]. Microvascular rarefaction, or decreased capillary density, is seen in both phenotypes but may be more functionally relevant in HFpEF, which contributes to impaired oxygen supply and energy mismatch. Cardiomyocyte hypertrophy is usually more noticeable in HFpEF [16]. Crucially, new data emphasize the role of right ventricular and left atrial remodeling in HFpEF, demonstrating the systemic character of the condition [13]. Furthermore, new evidence points to a possible involvement of unfolded protein response (UPR) activation and compromised protein quality control in the pathophysiology of HFpEF [15].

Systolic dysfunction, ventricular dilatation, and eccentric remodeling are the main symptoms of HFrEF, which is characterized by a substantial loss of cardiomyocytes as a result of ischemic injury, chronic pressure or volume overload, or genetic abnormalities [17]. A common characteristic of HFpEF is endothelial dysfunction, which tends to manifest earlier and frequently precedes overt heart failure, while in HFrEF it is more frequently a result of myocardial remodeling and damage [18]. Although inflammation is a major factor in both types, it is more frequently secondary to direct myocardial injury in HFrEF and closely related to metabolic and vascular comorbidities in HFpEF [13,14]. The “multiple phenotype hypothesis” emphasizes the heterogeneity of HFpEF even more by arguing that it is not a single disease process but rather the result of the convergence of multiple different pathophysiological pathways [13]. The observed disparities in clinical presentation, responsiveness to medication, and prognosis between HFpEF and HFrEF are caused by these mechanistic differences, underscoring the necessity of management techniques tailored to individual phenotypes [13,18].

### 1.4. Limitations of Existing Pharmacologic and Device Therapies

Despite improvements in the treatment of heart failure, HFrEF and HFpEF still pose significant therapeutic obstacles. When started early and in combination (referred to as “quadruple therapy”), there is strong evidence that guideline-directed medical therapy (GDMT)—which includes renin-angiotensin system inhibitors (RASi), angiotensin receptor-neprilysin inhibitors (ARNI), beta-blockers, mineralocorticoid receptor antagonists (MRA), and sodium-glucose cotransporter 2 inhibitors (SGLT2i)—reduces mortality and morbidity in HFrEF [19]. Less than 25% of patients, according to real-world data, reach the required dosages of all prescribed medication classes, and up to 30% stop treatment because of adverse effects such as hyperkalemia, hypotension, and renal failure [19]. Patient comorbidities, intolerance, and care inequities further hinder implementation, with lower-income areas having greater mortality and fewer access to cutting-edge treatments [20].

For some HFrEF patients, device therapies including implantable cardioverter-defibrillators (ICDs) and cardiac resynchronization therapy (CRT) offer extra benefits. However, not all patients are eligible or willing to have a device implanted, and response rates to CRT vary (30–40%) [19]. The situation is considerably more difficult with HFpEF. Due to aging, obesity, diabetes, and hypertension, HFpEF is becoming more common and now makes up more than half of all heart failure cases. Similarly to HFrEF, HFpEF patients have significant hospitalization rates (annual rate ~1.4 per patient) and a one-year mortality rate of 20–29% [20,21]. Large randomized trials produced neutral findings, and until recently, the majority of pharmacologic treatments, such as ACE inhibitors, ARBs, and MRAs, failed to demonstrate a meaningful mortality benefit in HFpEF [22]. SGLT2 inhibitors are the first class to consistently reduce hospitalizations for heart failure throughout the whole spectrum of ejection fraction; nonetheless, their impact on cardiovascular or all-cause mortality is still minimal [22]. According to the FINEARTS-HF trial, newer medications like finerenone have likewise not been able to significantly lower overall or cause-specific mortality in HFpEF [23]. There is no universally effective disease-modifying treatment for HFpEF, and its heterogeneity, driven by a variety of cardiac and non-cardiac comorbidities, complicates trial design and therapeutic targeting. Although they are being studied, device-based interventions such as interatrial shunt devices have not yet shown a definite long-term benefit [22].

The death and readmission rates of hospitalized patients with advanced HFpEF are comparable to those of patients with HFrEF, according to recent comparative studies [20]. Although sudden cardiac death and progressive heart failure are the primary causes of cardiovascular death in both HFpEF and HFrEF, individuals with HFpEF also bear a significant burden of non-cardiovascular mortality, which further reduces the effectiveness of cardiac therapy [23]. Thus, HFpEF remains a syndrome with significant unmet need, lacking strong, broadly applicable pharmacologic or device-based treatments, while HFrEF care is hindered by underutilization and side effects of proven medicines [20,21,22].

### 1.5. Rationale for Neuromodulation and the Emerging Role of AI

For both HFrEF and HFpEF, persistent gaps in pharmacologic and device-based therapy have prompted research into new strategies that focus on the underlying pathophysiology of heart failure. Neuromodulation, which attempts to address the autonomic imbalance that is essential to the development of both HFrEF and HFpEF and is typified by sympathetic overactivity and vagal withdrawal, is one of the most promising approaches [24,25]. Current treatments do not adequately address the negative effects of chronic sympathetic nervous system activation and decreased parasympathetic tone, which lead to arrhythmogenesis, systemic inflammation, and unfavorable cardiac remodeling [24,25]. A number of neuromodulatory methods have been created and tested in clinical settings. In animal models and human research, vagus nerve stimulation (VNS), baroreceptor activation therapy (BAT), renal sympathetic denervation (RDN), and low-level tragus stimulation (LLTS) have all shown promise in modifying autonomic tone, lowering inflammation, and enhancing cardiac function [26]. For example, a recent randomized, sham-controlled study showed that noninvasive LLTS significantly improved global longitudinal strain, decreased inflammatory cytokines, and improved quality of life in patients with HFpEF. These findings were not possible with conventional pharmaceutical treatments [26]. Likewise, BAT has been approved by the FDA for a limited number of patients after showing improvements in NT-proBNP levels, exercise capacity, and quality of life in HFrEF patients who continue to have symptoms despite receiving the best medication and device therapy. It is anticipated that ongoing pivotal trials like BeAT-HF and BiRD-HF will shed light on how BAT affects heart failure hospitalizations and mortality in advanced HFrEF [24]. In heart failure patients who are not responding to traditional pacing or defibrillation, cardiac neuromodulation therapy (CNT), administered via devices like the Moderato^®^ System, is also being researched for its potential to improve outcomes by lowering sympathetic activity [27].

Simultaneously, the field is changing quickly due to the incorporation of artificial intelligence (AI) into the treatment of heart failure. Artificial intelligence (AI) encompasses computational systems designed to perform tasks typically requiring human intelligence, including learning, problem-solving, and decision-making. Its rapid advancement holds significant promise across various medical disciplines, particularly in cardiology. Large-scale clinical and device data analysis, patient selection optimization for neuromodulation treatments, and real-time guidance during device insertion and follow-up are now all accomplished by AI-driven algorithms [25]. In order to maximize effectiveness and minimize side effects, machine learning algorithms can forecast individual risk profiles, identify phenotypes most likely to benefit from neuromodulation, and customize therapy. Additionally, AI-enabled analytics help close the gap left by traditional methods by enabling remote monitoring, early decompensation identification, and dynamic device parameter correction [25].

When combined, neuromodulation and AI offer mechanism-based, tailored therapies that address the ongoing unmet needs in both HFrEF and HFpEF, thereby bringing about a paradigm shift in the treatment of heart failure. These novel approaches have the potential to close the therapeutic gap that persists despite advancements in conventional pharmacologic and device therapies by directly regulating autonomic tone and utilizing advanced analytics for precision medicine [24,25,26].

The purpose of this review is to investigate the potential benefits of artificial intelligence (AI) in augmenting neuromodulation treatments for heart failure with preserved and reduced ejection fraction (HFpEF and HFrEF). We highlight the importance of AI in enhancing patient selection, therapy optimization, and real-time decision-making, concentrate on the causes underlying autonomic imbalance, and provide an overview of contemporary neuromodulation techniques. AI-guided neuromodulation has the potential to advance precision medicine by filling current therapeutic gaps. The facts, difficulties, and potential paths forward in this developing topic are all thoroughly covered in this review.

### 1.6. Methods

This review applied a structured literature search technique to ensure thorough coverage of current information on neuromodulation and artificial intelligence (AI) in heart failure therapy. Electronic databases such as PubMed, Embase, Scopus, and Web of Science were thoroughly searched for relevant papers published between January 2000 and December 2024. Search terms and Medical Subject Headings (MeSH) included combinations of: “heart failure,” “HFpEF,” “HFrEF,” “neuromodulation,” “autonomic modulation,” “vagus nerve stimulation,” “baroreflex activation therapy,” “spinal cord stimulation,” “cardiac contractility modulation,” “renal nerve denervation,” “artificial intelligence,” “machine learning,” “deep learning,” and “closed-loop systems.”

Manual searches of reference lists from relevant primary research and reviews were carried out to find additional publications. Two authors independently screened titles and abstracts, followed by a full-text review to determine eligibility. Discrepancies were handled by discussing or consulting with a third reviewer.

**Inclusion Criteria:**

1. Peer-reviewed original research publications, clinical trials, and review papers.
2. Studies with real subjects or appropriate preclinical models.
3. Articles on neuromodulation, autonomic modulation, and AI integration in heart failure.
4. Publications in English.

**Exclusion Criteria:**

1. Abstracts from conferences that are not available in full text.
2. Editorials, commentaries, or opinion pieces that lack original data.
3. Non-English language publications.
4. Studies not connected to cardiovascular disease or neuromodulation.

## 2. Autonomic Dysregulation in HFpEF and HFrEF

Autonomic nervous system (ANS) dysregulation is a pivotal contributor to heart failure (HF) pathophysiology and progression across its phenotypes: heart failure with preserved ejection fraction (HFpEF) and heart failure with reduced ejection fraction (HFrEF) [28]. It is primarily defined by a sympathovagal imbalance coupled with chronic neurohormonal activation. While stemming from cardiovascular injury, the specific autonomic patterns differ notably between HFrEF and HFpEF, impacting therapeutic approaches like neuromodulation [28,29].

### 2.1. Neurohormonal Activation in Heart Failure Progression

Neurohormonal activation, initially an adaptive response to cardiac injury or stress, becomes profoundly maladaptive when sustained, fueling a cycle of cardiac and systemic dysfunction [29].

#### 2.1.1. RAAS and SNS: Initial Compensatory Mechanisms

Following cardiac insult or reduced cardiac output, the sympathetic nervous system (SNS) and renin–angiotensin–aldosterone system (RAAS) are activated. SNS catecholamine release increases heart rate, contractility, and vasoconstriction to maintain perfusion. RAAS activation, via angiotensin II and aldosterone, promotes vasoconstriction and sodium/water retention, aiming to restore hemodynamics [29].

#### 2.1.2. Chronic Neurohormonal Overdrive and Maladaptation

Persistent SNS and RAAS activation becomes detrimental. Prolonged SNS hyperactivity increases myocardial oxygen demand and triggers pathological hypertrophy, apoptosis, and fibrosis. Chronic RAAS activation, particularly elevated aldosterone, promotes myocardial and vascular fibrosis and endothelial dysfunction. This sustained neurohormonal barrage exacerbates hemodynamic stress, systemic inflammation, and ventricular remodeling, leading to sympathovagal imbalance—augmented sympathetic and attenuated vagal tone that actively drives HF progression [29]. Early therapeutic intervention, before irreversible changes like extensive fibrosis occur, is crucial. The multifaceted damage underscores the rationale for combination therapies in HFrEF (e.g., beta-blockers, RAAS inhibitors, MRAs) targeting this neurohormonal onslaught [29].

### 2.2. Contrasting Autonomic Profiles in Heart Failure Phenotypes

While common, autonomic dysregulation manifests differently in HFrEF and HFpEF.

#### 2.2.1. HFrEF: Sympathetic Predominance

HFrEF is marked by profound adrenergic hyperactivity with increased sympathetic nerve discharge and loss of physiological sympathetic oscillations, independently predicting adverse prognosis [30]. This sympathetic overdrive is driven by deranged cardiovascular reflexes: blunted arterial baroreflex sensitivity (BRS), potentially paradoxical cardiopulmonary reflex responses, sensitized cardiac sympathetic afferent reflex (CSAR), and hyperactive arterial chemoreflexes. Chronic sympathetic activation itself worsens remodeling and baroreflex function, creating a vicious cycle. Thus, SNS and RAAS antagonism are foundational HFrEF therapy [30].

#### 2.2.2. HFpEF: Heterogeneous Syndrome with Vascular Stiffness and Chronotropic Incompetence

HFpEF also involves RAAS and SNS activation, though potentially less pronounced and more heterogeneous than in HFrEF; about 67% of patients show elevated neurohormonal biomarkers [31]. The pathophysiological role of this activation is less defined. While some HFpEF patients exhibit modest sympathetic overdrive (e.g., increased muscle sympathetic nerve activity), its primary role is debated, often being intertwined with highly prevalent comorbidities (hypertension, obesity, diabetes, age) that independently drive sympathetic activation [32].

More distinct autonomic-related features in HFpEF include significant vascular stiffness and chronotropic incompetence (CI). Increased central aortic stiffness elevates left ventricular systolic load and impairs ventricular-vascular coupling, especially in older, hypertensive women, and is a hallmark of HFpEF [33]. CI, an inadequate heart rate response to exercise, affects 30–50% of HFpEF patients, contributing to exercise intolerance and low peak VO2 [34]. CI mechanisms may involve impaired beta-adrenergic signaling or broader autonomic dysfunction, though intrinsic sinus node function often appear preserved, unlike in HFrEF [34]. Despite neurohormonal activation, traditional neurohormonal antagonists largely lack prognostic benefit in broad HFpEF populations (except perhaps HFmrEF), questioning the universal pathogenic dominance of cardiac sympathetic hyperactivity in this syndrome [31,32].

#### 2.2.3. Comparative Analysis of Neurohormonal and Autonomic Imbalance

**Figure 1:**
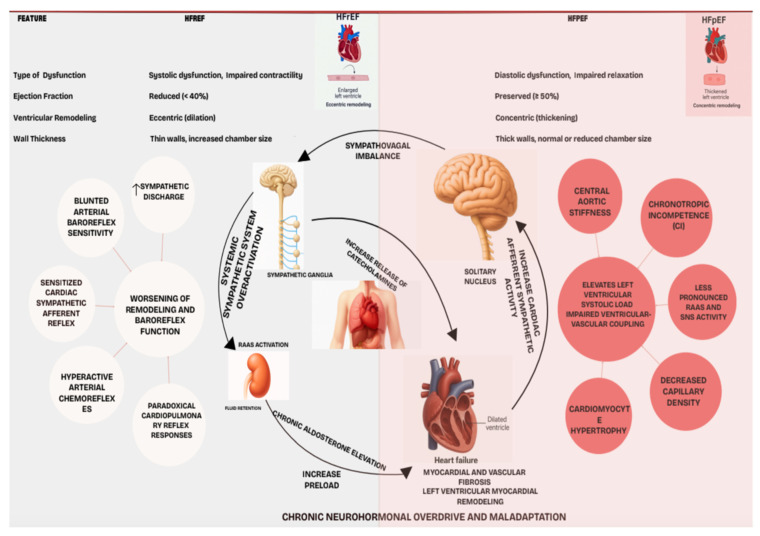
Pathophysiological model of autonomic dysregulation in heart failure, distinguishing phenotype-specific mechanisms.

Neurohormonal Activation is generally greater and more consistent in HFrEF, which exhibits clear sympathetic predominance and parasympathetic withdrawal [29,30]. HFpEF presents a more complex, heterogeneous, and potentially intermediate autonomic phenotype, often confounded by comorbidities that influence autonomic tone; sympathetic activation in HFpEF may be more related to these conditions than to an intrinsic HFpEF feature [32]. While reflex dysfunctions (baroreflex, chemoreflex) are well-documented in HFrEF, their role in HFpEF is less clear. Prominent CI and vascular stiffness are more distinguishing in HFpEF [33,34]. The differential response to neurohormonal blockade (effective in HFrEF, largely ineffective in HFpEF) implies fundamental differences in the pathophysiological dominance or nature of neurohormonal activation (Figure 1). In HFpEF, it might be secondary to drivers like inflammation or metabolic dysfunction, causing myocardial stiffening, or target tissues might respond differently. Distinct HFpEF traits (stiffness, CI) and less definitive evidence for profound global cardiac sympathetic hyperactivity suggest autonomic modulation targets in HFpEF may need to extend beyond simple sympathetic blockade. While global cardiac sympathetic hyperactivity is generally less pronounced in HFpEF compared to HFrEF, emerging evidence suggests that autonomic dysfunction in HFpEF is often heterogeneous and phenotype-specific. This dysfunction can manifest as chronotropic incompetence, impaired baroreflex sensitivity, and altered vascular tone contributing to increased arterial stiffness and elevated filling pressures. Therefore, rather than broad sympatholysis, the rationale for neuromodulation in HFpEF lies in its potential to precisely target these specific autonomic dysregulations.

AI-driven deep phenotyping becomes critical for dissecting this HFpEF heterogeneity. By identifying distinct patient subgroups based on their unique pathophysiological and autonomic profiles, AI can guide tailored neuromodulation interventions. For instance, in patients with chronotropic incompetence, interventions might focus on improving heart rate reserve, while in those with prominent vascular stiffness, therapies aimed at restoring endothelial function and modulating sympathetic outflow to the vasculature could be beneficial. This targeted approach moves beyond a ‘one-size-fits-all’ strategy, offering personalized neuromodulation to manage specific autonomic comorbidities and thereby improve exercise capacity and quality of life in select HFpEF populations [31,32].

### 2.3. Therapeutic Rationale for Targeting Autonomic Pathways

Autonomic dysregulation in both HF phenotypes warrants ANS-targeted therapeutic strategies, including neuromodulation, especially given residual risks with pharmacological interventions and HFpEF’s challenges [35].

#### 2.3.1. Restoring Sympathovagal Balance

A central goal is restoring physiological sympathovagal balance. In HFrEF, this primarily means attenuating sympathetic overdrive and augmenting vagal tone [35]. In HFpEF, objectives are more nuanced; while enhancing parasympathetic activity could be beneficial, modulating specific reflex pathways contributing to vascular stiffness or CI may be more relevant, as global cardiac sympathetic hyperactivity is less evident. Despite existing therapies, high morbidity/mortality, especially in advanced HF and HFpEF, calls for novel approaches like neuromodulation [35].

#### 2.3.2. Phenotype-Specific Interventions

Therapies should be tailored. For HFrEF’s sympathetically driven pathology, beta-blockade, BAT, and VNS aim to reduce sympathetic outflow and/or enhance parasympathetic influence [36]. For heterogeneous HFpEF, optimal targets are less defined. Interventions might focus on:

- Improving Chronotropic Responsiveness: Addressing impaired beta-adrenergic sensitivity or central autonomic control [34].
- Reducing Stiffness: Modulating sympathetic vascular outflow or other ANS pathways affecting vascular compliance [33].
- Managing Autonomic Comorbidities: Targeting conditions like hypertension or sleep apnea.

The failure of broad neurohormonal antagonists in unselected HFpEF suggests that precise, potentially AI-guided neuromodulation, personalized to specific dysfunctions (e.g., CI, vascular stiffness subtypes) rather than global sympatholysis, is needed. Deep phenotyping could guide such targeted therapies, potentially revolutionizing HFpEF treatment [35]. Table 1 provides comparative summary of autonomic dysregulation in HFrEF and HFpEF.

## 3. Neuromodulation Modalities: Mechanisms and Clinical Data

Despite advances in heart failure management, including quadruple therapy and cardiac resynchronization therapy, morbidity and mortality remain substantial. Therefore, a need is being felt to explore other treatment modalities alongside pharmacologic therapy [19,21,35]. The Autonomic Nervous System (ANS) plays a major role in the pathophysiology of heart failure. The sympathetic and parasympathetic nervous systems interact with each other, along with regional responses and feedback from the central nervous system, to influence cardiac function [28]. Acute sympathetic activation helps maintain cardiac output, but chronic sympathetic stimulation in heart failure patients increases cardiac stress, leading to myocyte enlargement and ventricular remodeling [29,30]. Recognizing the ANS’s critical role, neuromodulation techniques are being developed that directly modulate its activity through electrical or chemical stimuli [24]. The various modalities currently under study and proving successful are detailed below:

### 3.1. Vagus Nerve Stimulation (VNS)

**PRINCIPLE:** Heart failure primarily leads to autonomic imbalance with sympathetic overactivation and parasympathetic underactivation [28,37]. The primary goal of VNS is to increase parasympathetic tone by stimulating the vagus nerve [38].

**METHODS:** VNS involves surgically implanting a device, typically by placing a standard transvenous lead into the right ventricle and a nerve stimulation cuff on the vagus nerve. The leads are then tunneled and connected to a pulse generator [39,40].

**TRIALS:** Various studies and randomized controlled trials have tested the efficacy and safety of VNS, notably ANTHEM-HF, NECTAR-HF, and INOVATE-HF, summarized in Table 2.

**SAFETY:** The safety profile for VNS in heart failure appears acceptable, with an overall infection rate comparable to that in patients implanted with a VNS system for epilepsy treatment [41,42]. Reported side effects include oropharyngeal pain, implant site pain, voice alteration, and hoarseness [40]. The primary safety endpoints were met in all studies, rendering the device generally safe and durable.

**EFFICACY:** The ANTHEM-HF study showed promising results, with significant improvements in LVEF, LVESD, NYHA Class, 6 min walk distance, heart rate variability, and MLHFQ score after 6 months of therapy (*p* < 0.005) [40]. However, Vagus nerve stimulation has also been investigated in pivotal trials with neutral or negative outcomes, notably NECTAR-HF and INOVATE-HF. The **NECTAR-HF Study** failed to demonstrate a significant improvement in left ventricular (LV) remodeling parameters or LV function following 6 months of VNS therapy [41]. Similarly, the **INOVATE-HF Study** revealed that VNS therapy did not reduce the composite rate of death or heart failure events in patients with chronic heart failure with reduced ejection fraction [39].

The neutral or negative outcomes observed in these trials have prompted considerable discussion regarding the underlying reasons. A key suspected factor is the suboptimal, heuristic approach to VNS parameter determination, which largely relied on open-loop, manual titration. This method may not have adequately accounted for the significant inter-individual variability in autonomic responses to VNS. Consequently, it is hypothesized that the therapy parameters used might not have been optimally tuned for efficacy in a broad patient population. This highlights a crucial area for future research, suggesting that more sophisticated parameter optimization strategies are needed to accurately evaluate the true efficacy of VNS in future heart failure trials [39,41].

### 3.2. Baroreceptor Activation Therapy (BAT)

**PRINCIPLE:** Baroreceptors are stretch-sensitive mechanoreceptors located in the carotid sinus and the aortic arch, innervated by both the parasympathetic and sympathetic nervous systems [43]. Baroreceptor sensitivity, a measure of the ANS’s ability to regulate blood pressure, is impaired in chronic heart failure patients [44]. BAT targets this pathophysiologic disturbance.

**METHODS:** The BAT setup consists of a carotid sinus lead and a pulse generator. It involves placing a 2 mm electrode on the carotid sinus and connecting it to a subcutaneously implanted pulse generator, which stimulates at a set device setting [45].

**TRIALS (BeAT-HF):** BeAT-HF (Baroreflex Activation Therapy for Heart Failure) was a prospective, multicenter, randomized controlled trial. It enrolled 408 patients (NYHA Class II/III, LVEF ≤ 35%, 6 min walk distance 150–400 m, on stable guideline-directed medical therapy for ≥1 month), randomized 1:1 to receive either BAT plus optimal medical therapy or only maximally tolerated guideline-directed medical therapy [36,45].

**SAFETY:** This device was found to be safe in the trial; an adverse event was reported in 7 patients who underwent BAT within 30 days post-implantation, with no additional events noted until 6 months [36].

**EFFICACY:** BeAT-HF is considered the first successful trial of a device-based neuromodulation for heart failure with reduced ejection fraction. BAT is safe in HFrEF patients and significantly improves exercise capacity and functional status (*p* < 0.005) [36].

### 3.3. Spinal Cord Stimulation (SCS)

**PRINCIPLE:** SCS has long been used for neuropathic pain and non-revascularizable angina [46,47]. Its mechanism may involve modulating sympathetic tone and/or vagal stimulation [48,49]. Some studies suggest association with vasodilatory effects and decreased catecholamine production [50].

**METHODS:** SCS involves inserting a single lead with 8 electrodes into the epidural space at the thoracic level by a neurosurgeon, with typical stimulation parameters of 50 Hz frequency and 0.2 ms duration [51,52].

**TRIALS:**

- **DEFEAT-HF** (Determining the Feasibility of Spinal Cord Neuromodulation for the Treatment of Heart Failure) [52]: Stimulation at T2-4 level for 12 h/day. Primary endpoint was difference in left ventricular end-systolic volume index after 6 months. **Results:** Thoracic (T2-4) SCS did not lead to changes in LV structural remodeling at 6 months.
- **SCS-HEART** (Spinal Cord Stimulation for HF) [53]: Stimulation at T1-3 level continuously. **Results:** High thoracic SCS can lead to improvements in LV function and exercise tolerance.

**SAFETY:** This device is generally reported as safe and well-tolerated. Reported adverse events include lead dislodgement, implant site hematoma, and decompensated heart failure, occurring in less than 5% of patients [52].

**EFFICACY:** The main difference between the two trials was the stimulation areas, suggesting that frequency and intensity of stimulation, along with the stimulated area, can affect outcomes (*p* < 0.005) [38].

### 3.4. Renal Nerve Denervation (RND)

**PRINCIPLE:** In heart failure patients, renal nerves are overactive. RND aims to eliminate the sensory afferent and sympathetic efferent fibers of the renal nerve [54].

**METHODS:** RND is accomplished by surgically stripping the renal nerves or by non-invasively ablating the renal artery [38].

**TRIALS:**

- **REACH-HF** [55]: A prospective, double-blinded, randomized, controlled study including 7 patients with chronic systolic heart failure on maximal tolerated therapy.
- **SYMPLICITY-HF** [56]: Enrolled 39 patients with chronic systolic HF (LVEF < 40%), NYHA class II-III, and renal impairment on stable medical therapy.

**SAFETY:** RND was found to be safe, with no peri- or post-procedural side effects noted in REACH-HF [55]. One patient reported renal artery occlusion in SYMPLICITY-HF [56].

**EFFICACY:** Results suggested improvements in both symptoms and exercise capacity in the REACH-HF trial (*p* < 0.005) [38,55]. However, the SYMPLICITY-HF trial showed no improvement in heart function at 12 months following renal denervation therapy, though reports showed a reduction in NT-proBNP [38,56].

### 3.5. Cardiac Sympathetic Denervation (CSD)

**PRINCIPLE:** CSD focuses on reducing the activity of the sympathetic nervous system.

**METHODS:** This is accomplished by surgically removing the lower half of the stellate ganglion through the T2-T4 thoracic ganglia [56]. This eliminates both afferent signaling and sympathetic postganglionic efferents to the heart. Videothoracoscopic clipping is performed under general anesthesia with single-lumen endotracheal intubation [57].

**TRIALS:** CSD has been used as an antiarrhythmic therapy for severe arrhythmias, and its efficacy has recently been studied in heart failure patients. A pilot study evaluated left cardiac sympathetic denervation as a potential treatment for symptomatic systolic heart failure. 15 patients were chosen, randomized 2:1, with 10 receiving LCSD plus medical therapy and 5 receiving medical therapy alone [57].

**SAFETY:** The incidence of minor complications was in the acceptable range, including slight sweating in the plantar area and thoracic pain relieved with analgesics. Possible complications include Horner’s syndrome [56,57].

**EFFICACY:** The pilot study resulted in improvement of clinical status, NYHA class, 6 min walking distance, and other measures (*p* < 0.005). The main limitation of this study was its very small sample size [57].

### 3.6. Cardiac Contractility Modulation (CCM)

**PRINCIPLE:** The basis of cardiac contractility modulation formed from observations in 1969 that excitatory stimulation applied during the absolute refractory period augments myocardial contraction [58].

**METHODS:** CCM devices are implanted similarly to a permanent pacemaker and ICD [59]. Neuromodulation is accomplished using standard pacing electrodes to deliver a biphasic impulse of 7.5 volts for 22 ms to the right ventricular septum during the absolute refractory period, for 5–12 h per day. These non-excitatory electrical signals enhance the strength of cardiac muscle contraction [60].

**TRIALS:**

- **FIX-HF-4** [61]: A double-blinded, prospective, double-crossover study conducted in Europe; 164 patients were randomized. Patients with heart failure on guideline-directed medical therapy received 12 weeks of CCM.
- **FIX-HF-5** [62]: A prospective randomized controlled trial studying CCM efficacy in patients with NYHA III/IV, EF ≤ 35%. 428 patients with narrow QRS heart failure were enrolled and randomized to optimal medical therapy and CCM vs. optimal medical therapy alone.
- **FIX-HF-5C** [63]: A confirmatory study for the FIX-HF-5 trial, this was a prospective, randomized study including 160 people randomized 1:1 to receive optimal medical therapy and CCM vs. optimal medical therapy alone.

**SAFETY:** Cardiac contractility modulation devices were found to be safe and generally well-tolerated in all three trials [61,62,63].

**EFFICACY:** Significant improvement in peak VO2 was seen in the FIX-HF-4 trial (*p* < 0.005). The primary efficacy endpoint was achieved in both FIX-HF-4 and FIX-HF-5C trials, but not in the FIX-HF-5 trial. The secondary endpoints were met in the FIX-HF-5 study, prompting the confirmatory study [61,62,63].

### 3.7. Emerging Neuromodulation Modalities

**TRAGUS NERVE STIMULATION:** This is a non-invasive way of stimulating the vagus nerve, as its auricular branch is connected to the skin of the tragus [64]. The ability of this neuromodulation modality to preferentially activate afferent rather than efferent vagal fibers leads to more significant inhibition of sympathetic activity [65]. It has also been shown that tragus stimulation can reduce the burden of atrial fibrillation [66].

**ENDOVASCULAR BAROREFLEX ACTIVATION:** This aims to reduce sympathetic overactivity. A self-expanding stent is implanted in the internal carotid artery, which changes the geometric shape of the carotid sinus, thereby augmenting the baroreflex mechanism [67].

**PHRENIC NERVE STIMULATION:** This is a newer modality that aims to treat patients with central sleep apnea and heart failure, which has a poor prognosis. The remedē^®^ System has a lead and pulse generator that stimulates the phrenic nerve, causing contraction of the diaphragm similar to normal breathing. It automatically stimulates the phrenic nerve when the patient is sleeping at night. It detects apneic episodes, thereby reducing the overload on the heart [68].

## 4. Role of Artificial Intelligence in Cardiovascular and Heart Failure Care

Building upon its foundational capabilities, Artificial Intelligence (AI) holds immense theoretical and preclinical potential across various aspects of cardiovascular care, leveraging its subfields such as machine learning (ML) and deep learning (DL). These advanced analytical methods are transforming how complex clinical data are processed and interpreted for heart failure management [69]. AI can make decisions without human intervention and is programmed to do so independently through various algorithms and human calculations [69,70]. While promising, many of these advanced AI applications are currently explored in computational models or preclinical animal studies, with clinical integration still in nascent stages.

### 4.1. Machine Learning (ML)

Machine Learning (ML) is a subset of artificial intelligence defined as a technique that analyzes data without being explicitly programmed [71]. It identifies and learns patterns from given data, then applies them to new data and performs computational tasks, often with superior efficiency to a fatigable human mind [71,72]. There are three representative methods in machine learning [71,72,73]:

Supervised machine learning: Uses human-labeled datasets to train algorithms for correct data classification and outcome prediction.

Unsupervised machine learning: Uses unlabeled datasets and independently identifies hidden patterns in the data.

Semi-supervised machine learning: Combines both supervised and unsupervised machine learning, using both labeled and unlabeled datasets for training.

### 4.2. Deep Learning (DL)

Deep learning is a subset of machine learning that mimics the functioning of the human brain [70]. It uses multiple layers of artificial neural networks to discover and predict patterns [70,74]. The neural networks are organized into an input layer and an output layer. When data is received by the neural network, the input layer becomes active and transmits signals ultimately to the output layer [70].

### 4.3. Reinforcement Learning (RL)

Reinforcement learning is a type of machine learning that learns by trial and error to earn a reward [75]. The primary aim of the agent is to perform actions that maximize positive rewards and minimize negative rewards [75].

### 4.4. General Applications of Artificial Intelligence in Heart Failure

Prediction of Heart Failure: Machine learning can predict the possibility of future heart failure based on risk factors, even identifying new, previously unconsidered risk factors. This allows for early counseling, treatment, and follow-up for at-risk individuals to reduce disease severity and symptom chances [76].

Diagnosis of Heart Failure: Algorithms like DEHF (DL algorithm for ECG-based HF identification) have been developed for early heart failure diagnosis based on electrocardiograms, demonstrating superior detection capabilities [77]. AI-CDSS (AI-Clinical Decision Support System) also shows remarkable diagnostic accuracy for heart failure [78]. These algorithms can facilitate early diagnosis and prompt initiation of optimal medical management, potentially preventing ventricular remodeling due to chronic heart failure [76].

Better Classification of Heart Failure Phenotypes: Machine learning can enhance heart failure classification through phenotype mapping, utilizing algorithms and electronic health record data [70]. This aids in understanding different phenotypes and their response to treatment [79].

Prognosis and Prediction of Outcomes Following Diagnosis of Heart Failure: Accurate risk prediction is possible with machine learning. The DAHF (DL algorithm for predicting the mortality of patients with Acute HF) is an AI model for risk-stratifying patients [80]. Data from cardiac monitoring and vitals can further guide this process.

Optimization of Personalized Medical and Device Therapy (General): Machine learning can assist in prescribing correct guideline-directed medical therapy and identifying patients at risk for side effects [81]. It can also facilitate patient selection for general device therapies like CRT or ICD, by detecting certain parameters to predict response [82].

## 5. AI-Guided Neuromodulation: Concept and Implementation

AI holds immense theoretical and preclinical potential to revolutionize neuromodulation in heart failure by enabling highly personalized and effective therapies. While promising, many advanced AI applications are currently explored in computational models or preclinical animal studies, with clinical integration still in nascent stages. It is important to note that AI-guided parameter optimization and closed-loop control are not yet standard practice in clinical heart failure neuromodulation, but represent areas of significant future integration potential.

### 5.1. AI-Driven Patient Selection for Neuromodulation

**Figure 2:**
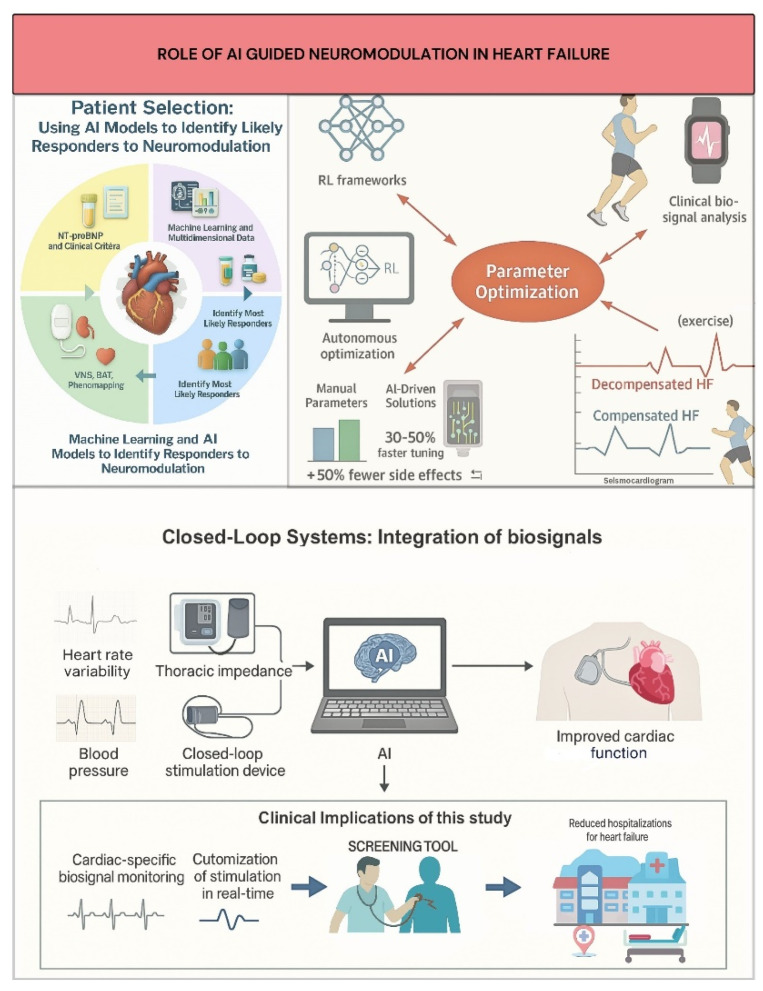
AI roles in neuromodulation: patient selection, parameter optimization, and adaptive control.

Effective patient selection is critical for neuromodulation therapy given the variability of disease morphologies and varied response rates to device-based interventions [25,36,83]. Traditional physician judgment and conventional clinical criteria often fall short in capturing the intricate interactions affecting treatment results. Machine learning (ML) and Artificial Intelligence (AI) models have become effective instruments for integrating and analyzing large-scale, multidimensional datasets, such as imaging, laboratory biomarkers, electronic health records (EHRs), and device telemetry, to determine which patient subgroups are most likely to react to neuromodulation (Figure 2) [27,84].

For example, ML-based phenomapping has allowed for more accurate targeting of treatments like Vagus Nerve Stimulation (VNS) and Baroreflex Activation Therapy (BAT) by classifying heart failure patients into discrete clusters with varying autonomic dysfunction and inflammatory profiles [79,85]. While patient selection based on NT-proBNP and clinical factors was crucial in the pivotal BeAT-HF study, new AI techniques seek to further hone these criteria, aiming to increase responder prediction accuracy [27,86]. AI tools are also being developed to rapidly screen electronic health records for clinical trial eligibility, potentially accelerating enrollment and reducing costs. Furthermore, AI algorithms analyzing continuous data from implanted devices and wearable sensors show promise in early detection of autonomic imbalance and impending decompensation, enabling timely neuromodulation intervention and dynamic, personalized patient assessment [24,83]. AI analyzing accessible data (e.g., single-lead electrocardiograms) can estimate heart failure risk, aiding candidate selection for interventions like neuromodulation [86].

### 5.2. AI for Parameter Optimization and Closed-Loop Systems

Optimizing stimulation parameters (amplitude, pulse width, frequency) is a major hurdle for translating neuromodulation, as traditional open-loop, manual titration is often suboptimal and time-consuming. This heuristic approach may not adequately account for significant inter-individual variability in autonomic responses, potentially explaining neutral outcomes in some trials (e.g., NECTAR-HF, INOVATE-HF for VNS) [39,41].

Closed-loop neuromodulation devices constitute a major advancement, allowing dynamic, real-time stimulation parameter adjustment based on ongoing cardiac-specific biosignal monitoring [25,86]. Unlike fixed-parameter open-loop devices, these platforms incorporate physiological markers like blood pressure, thoracic impedance, and heart rate variability (HRV) to customize autonomic modulation to the patient’s current hemodynamic and autonomic status, increasing efficacy and reducing side effects (Figure 2) [25,87]. For example, BAT systems, while currently physician-programmed, present opportunities for AI to personalize initial settings based on patient data/biomarkers, and enable adaptive closed-loop therapy responsive to real-time physiological signals [87]. AI-driven solutions like HeartLogic™, which incorporates wearable sensor data (such as thoracic impedance and heart rate variability), can predict heart failure exacerbations with 70% sensitivity, supporting clinical applications and allowing for proactive therapy modifications [88]. Similarly, machine learning models using wearable seismocardiogram data during exercise have shown 89% accuracy in differentiating between compensated and decompensated heart failure conditions to determine optimal stimulation timing (Figure 2) [88]. Theoretical frameworks modeled after biological neuromodulatory systems (e.g., acetylcholine/noradrenaline pathways) suggest lifetime RL algorithms to adjust stimulation parameters to fluctuating HF conditions, ensuring continuous efficacy in non-stationary situations [89].

By combining clinical data and real-time biosignals, AI algorithms now allow for dynamic, patient-specific modification of these parameters. Computational studies use reinforcement learning (RL) to develop closed-loop VNS control frameworks, enabling real-time optimization of physiological parameters such as heart rate and blood pressure [90,91]. Machine learning models, such as long short-term memory networks, can map VNS parameters to physiological changes and integrate into control frameworks for real-time optimization [91].

These developments highlight AI’s potential to replace static, one-size-fits-all neuromodulation methods with precise, adaptive titration strategies that optimize therapeutic effectiveness and safety.

### 5.3. Preclinical Foundations of AI-Guided Neuromodulation

Animal models have been crucial for neuromodulation proof-of-concept in heart failure and are increasingly used for developing AI-guided and adaptive control strategies. Optimizing stimulation parameters is a major hurdle for translating neuromodulation, and AI-driven closed-loop systems are a key area of interest [90]. Computational studies utilizing reinforcement learning develop closed-loop VNS control frameworks, enabling real-time optimization of physiological parameters like heart rate and blood pressure [91]. Machine learning models (e.g., long short-term memory networks) can map VNS parameters to physiological changes and integrate into control frameworks for real-time optimization [91]. Efficient neural modeling using machine learning-based surrogate models allows for rapid prediction of neural responses to stimulation, facilitating the design of selective VNS [92]. Developing robust HFpEF animal models (e.g., murine “2-hit” or “4-hit” protocols) is vital for testing advanced therapies, including AI-guided neuromodulation [93].

### 5.4. Translational Aspects, Future Directions, and Challenges

Translating neuromodulation to human heart failure has seen successes and setbacks, and current limitations highlight AI’s future integration potential. AI-enabled remote monitoring tools, such as the HeartLogic™ system, and analytics from non-invasive monitoring (e.g., in the LINK-HF2 study), provide real-world human data that can inform adaptive neuromodulation strategies [36]. Clinicians responded to AI notifications in a pilot study of the LINK-HF2 trial, guiding main trial implementation and demonstrating feasibility of integrating AI analytics into clinical workflows [36].

Neuromodulation research has predominantly focused on HFrEF, leaving a critical knowledge gap for HFpEF, where dedicated studies, particularly with AI-driven adaptive approaches, are scarce. While HFrEF allows AI to optimize personalized parameter selection, closed-loop adaptive systems, and responder identification, HFpEF’s heterogeneous nature presents greater challenges. For HFpEF, AI is an essential enabling technology for deep phenotyping, targeting specific mechanisms, and integrating comorbidity management [93].

Assessing neuromodulation efficacy uses standard clinical endpoints (e.g., functional capacity, quality of life, NYHA class) and biomarkers (e.g., NT-proBNP, noradrenaline, inflammatory markers, autonomic indices). AI and machine learning can personalize outcome prediction by integrating multi-modal data to find predictive patterns, aiding in patient selection and therapy adjustment.

While these developments hold promise for improving response rates and overcoming the drawbacks of previous fixed-parameter devices, issues with biomarker selection, algorithm robustness, and long-term clinical validation still exist. More research and extensive, sham-controlled trials are essential to prove closed-loop neuromodulation’s safety, effectiveness, and affordability as a pillar of precision medicine in the treatment of heart failure [25,89].

## 6. Preclinical and Clinical Evidence for Autonomic Neuromodulation in Heart Failure

Animal models have been crucial for neuromodulation proof-of-concept in heart failure (HF) and continue to inform therapeutic strategies. Translation to human heart failure has seen both successes and setbacks, with key lessons learned from various clinical trials.

### 6.1. Preclinical Foundations of Neuromodulation

#### 6.1.1. Vagus Nerve Stimulation (VNS) in Animal HF Models

Chronic VNS shows therapeutic potential in animal HF models (e.g., rat myocardial infarction, canine pacing). Benefits include improved left ventricular (LV) function (e.g., left ventricular ejection fraction, fractional shortening), reduced adverse remodeling, and improved survival [94]. Early studies used fixed VNS parameters, but individualized approaches are emerging. For instance, titrating VNS to achieve specific heart rate reductions (e.g., 20–30 beats per minute) improved cardiac function [94]. “Reactive VNS” (e.g., early post-myocardial infarction) and spatially selective VNS targeting specific vagal fibers to maximize therapeutic effects and minimize side effects are also being explored [95].

#### 6.1.2. Other Preclinical Neuromodulation Research

Spinal cord stimulation (SCS) improved left ventricular ejection fraction and reduced serum norepinephrine and brain natriuretic peptide in pigs with ischemic HF [96] and alleviated cardiac pain in rat myocardial infarction models via spinal microglial deactivation [97]. Baroreflex activation therapy (BAT) studies in animals showed that carotid baroreceptor stimulation reduced sympathetic activity and augmented parasympathetic signaling, providing the rationale for human heart failure with reduced ejection fraction (HFrEF) trials. Developing clinically relevant heart failure with preserved ejection fraction (HFpEF) animal models (e.g., murine “2-hit” or “4-hit” protocols) to replicate diastolic dysfunction, LV hypertrophy, and exercise intolerance are ongoing [98]. Robust HFpEF models are vital for testing advanced therapies.

### 6.2. Translation to Human Heart Failure: Clinical Trials

#### 6.2.1. VNS Clinical Trials: Key Findings

VNS has been extensively studied in HFrEF (LVEF ≤ 35−40%; e.g., NECTAR-HF, ANTHEM-HF, INOVATE-HF). A meta-analysis of 1263 HFrEF patients showed that VNS improved New York Heart Association (NYHA) class, quality of life (Minnesota Living with Heart Failure Questionnaire), 6 min walk test (6MWT), and N-terminal pro-brain natriuretic peptide (NT-proBNP) levels, with no difference in all-cause mortality and approximately 11% VNS-related adverse effects [92,99].

However, individual large randomized controlled trials had varied, sometimes inconclusive results, often failing primary efficacy endpoints. While the ANTHEM-HF trial demonstrated significant benefits in left ventricular remodeling and clinical status in patients with chronic heart failure with reduced ejection fraction using a specific VNS device [40], other major trials, such as NECTAR-HF [41] and INOVATE-HF [39], did not meet their primary endpoints. For instance, the NECTAR-HF trial failed to show significant improvement in LV end-systolic volume or clinical events with chronic VNS. A key differentiating factor in these neutral trials often centered on fixed or sub-optimally titrated stimulation parameters and open-loop device designs, suggesting that the efficacy of VNS is highly dependent on precise, individualized stimulation delivery.

#### 6.2.2. Baroreflex Activation Therapy (BAT) in HFrEF

BAT, which electrically stimulates carotid baroreceptors to decrease sympathetic activity and increase parasympathetic activity, has shown promise in HFrEF [36]. The efficacy and safety of BAT were rigorously evaluated in the large-scale randomized controlled trial (RCT), BeAT-HF. This robust clinical trial, which included 264 patients with heart failure with reduced ejection fraction, demonstrated statistically significant improvements in quality of life and exercise capacity [36]. The BeAT-HF trial (HFrEF, NYHA II/III, LVEF ≤ 35%, optimal medical management) found BAT safe and significantly improved quality of life, 6MWT distance, and NT-proBNP at 6 months versus medical therapy alone [36]. These quality-of-life benefits were durable at 24 months [36].

### 6.3. Comparative Analysis of Neuromodulation Evidence Across HFpEF and HFrEF Populations

Neuromodulation research has predominantly focused on HFrEF, often defined by LVEF ≤ 35−40%. This reflects HFrEF’s historically better-understood pathophysiology, especially sympathetic overactivity as a clear therapeutic target. Dedicated neuromodulation studies for HFpEF are scarce. While HFpEF animal models are improving [98], their use in testing advanced neuromodulation is nascent. HFpEF’s less defined autonomic profile makes direct translation of HFrEF neuromodulation strategies challenging.

### 6.4. Interpretation of Outcome Metrics and Biomarker Trends in Neuromodulation Studies

Assessing neuromodulation efficacy employs various clinical endpoints and biomarkers.

#### 6.4.1. Key Clinical Endpoints

Standard HF neuromodulation trial endpoints include functional capacity (6MWT), quality of life (Minnesota Living with Heart Failure Questionnaire), NYHA class, and morbidity/mortality. Improvements are seen with VNS [99] and BAT [45], though mortality benefits are not always observed.

#### 6.4.2. Biomarker Dynamics

NT-proBNP: Reductions are common in successful neuromodulation.

Noradrenaline: Levels often decrease with sympathetically attenuating neuromodulation.

Inflammatory Markers: VNS can reduce inflammatory cytokines.

Autonomic Indices: Heart rate variability and muscle sympathetic nerve activity can show improved autonomic balance.

#### 6.4.3. AI-Driven Personalized Outcome Prediction

AI and machine learning can personalize outcome prediction by integrating multi-modal data to find predictive patterns [100], aiding in patient selection and therapy adjustment. Table 3 shows major clinincal trials for heart failure and relevance to AI integration.

## 7. Discussion

Heart failure (HF) remains a major public health challenge, despite advances in medical therapy. Even though many studies have shown evidence of better outcomes through guideline-directed medical treatments, many patients continue to experience symptoms, hospitalizations, and reduced quality of life. Most research has concentrated on pharmacological interventions and structural heart treatments, with little consideration given to how the autonomic nervous system affects heart failure progression. Neuromodulation, which targets this system, has shown potential in early studies but has not been widely adopted in clinical practice. In addition, artificial intelligence (AI) is increasingly being used in cardiology, but its integration into neuromodulation strategies for heart failure is still a developing field. This study addresses these gaps by reviewing current neuromodulation techniques and examining how AI can enhance their effectiveness in managing both HFrEF and HFpEF.

This review emphasizes the growing role of neuromodulation as a promising adjunctive therapy in heart failure management. Techniques such as vagus nerve stimulation (VNS), baroreceptor activation therapy (BAT), spinal cord stimulation (SCS), and cardiac contractility modulation (CCM) have demonstrated varying degrees of clinical benefit, particularly in patients with heart failure with reduced ejection fraction (HFrEF). For example, the ANTHEM-HF and BeAT-HF trials reported improvements in left ventricular function, exercise tolerance, and symptom burden following neuromodulatory interventions [36,40]. While some modalities, such as SCS and renal nerve denervation, showed mixed or limited success in earlier trials (e.g., DEFEAT-HF), their mechanisms remain biologically plausible and require further investigation [52]. Baroreflex Activation Therapy (BAT) has been rigorously evaluated in pivotal randomized controlled trials. The BeAT-HF trial, a prospective, multicenter, randomized controlled study definitively demonstrated significant improvements in quality of life, exercise capacity, and heart failure hospitalization rates in patients with heart failure with reduced ejection fraction.

In contrast, Cardiac Sympathetic Denervation (CSD), while conceptually appealing, is still in earlier stages of clinical investigation. Pilot studies involving small cohorts (e.g., a study with 15 patients) have suggested beneficial hemodynamic and symptomatic effects in select patients with advanced heart failure. These preliminary findings are promising and warrant further investigation through larger, controlled trials to establish efficacy and safety. Additionally, we found that artificial intelligence can support these interventions by enhancing patient selection, optimizing device parameters, and predicting therapeutic response. Together, these findings suggest the potential for AI-assisted neuromodulation to complement guideline-directed medical therapy in both HFrEF and HFpEF populations.

The findings of this review support and expand upon prior research into neuromodulation in the treatment of heart failure. For instance, the ANTHEM-HF trial demonstrated significant improvements in left ventricular ejection fraction (LVEF) following vagus nerve stimulation, a result consistent with prior mechanistic studies that suggested parasympathetic enhancement may counteract sympathetic overdrive in HFrEF patients [40]. On the other hand, the NECTAR-HF trial reported limited structural benefits, indicating potential differences in patient selection and device programming [41]. Similarly, the BeAT-HF trial validated the efficacy of baroreceptor activation therapy, complementing previous reports that linked baroreflex impairment with disease progression in heart failure [36]. While the DEFEAT-HF trial did not show significant changes in cardiac remodeling with spinal cord stimulation, more recent data from SCS-HEART suggests this approach may still improve functional capacity when stimulation parameters are optimized [52,53]. These variations highlight the need for personalized neuromodulatory strategies. Furthermore, the integration of artificial intelligence into these therapies presents a novel dimension, as AI algorithms are increasingly being used to refine patient selection and predict therapeutic outcomes, as noted in recent reviews by Yoon et al., 2024 and Brunckhorst et al., 2006 [58,76]. Collectively, our review supports the hypothesis that AI-guided neuromodulation could supplement conventional pharmacologic therapy and fill existing gaps in treatment response among diverse heart failure subtypes.

Despite the insightful information gained from this review, several limitations must be acknowledged. First, many of the clinical trials included such as ANTHEM-HF, NECTAR-HF, and DEFEAT-HF had relatively small sample sizes and limited follow-up durations, which may affect the generalizability of their findings [40,41,52]. Additionally, heterogeneity in trial designs, patient selection criteria, and device parameters may have introduced variability that complicates direct comparisons across studies. For emerging neuromodulatory modalities like renal nerve denervation and phrenic nerve stimulation, the evidence base remains preliminary, with few large-scale randomized trials available. Furthermore, while artificial intelligence holds substantial promise in optimizing heart failure care, most applications remain in the early investigational phase and are not yet routinely integrated into clinical decision-making. These limitations suggest the need for more standardized protocols and long-term, multicenter studies to validate the durability and safety of these neuromodulatory interventions.

While this review emphasizes the growing role of neuromodulation and artificial intelligence in managing heart failure, further research is essential to refine and validate these approaches. Large-scale, multicenter randomized controlled trials with standardized methodologies are needed to better assess the long-term safety and efficacy of devices such as vagus nerve stimulators, baroreceptor activation systems, and spinal cord stimulators. Furthermore, as AI-based tools keep evolving, future studies should focus on integrating machine learning models into clinical workflows, ensuring transparency, accuracy, and equity in patient outcomes. It would also be beneficial to explore the combined use of AI-guided neuromodulation and pharmacologic therapy, which may offer synergistic results. Ongoing investigations into the molecular and autonomic mechanisms underlying both HFrEF and HFpEF will help in developing targeted and personalized treatment strategies.

## 8. Challenges and Future Directions

### 8.1. Challenges and Limitations

The integration of neuromodulation and artificial intelligence (AI) in managing heart failure (HF), particularly heart failure with preserved ejection fraction (HFpEF), faces significant challenges. One major limitation is the limited data available for HFpEF populations concerning neuromodulation and AI integration. While heart failure with reduced ejection fraction (HFrEF) has been more thoroughly researched, the distinct pathophysiology of HFpEF complicates the development of evidence-based protocols, hindering tailored treatment strategies. Additionally, technical barriers pose substantial obstacles, including the need for data standardization across diverse healthcare systems, real-time analytics to support timely clinical decisions, and effective sensor integration for continuous patient monitoring. These technical challenges make it difficult to deploy AI-driven neuromodulation solutions effectively. Furthermore, regulatory and ethical considerations, such as ensuring algorithm transparency, protecting data privacy, and resolving liability issues for AI-driven treatment errors, remain critical hurdles. Economic and infrastructural constraints also limit progress, as the high costs of developing advanced neuromodulation devices and AI platforms, combined with inadequate access to high-speed internet or advanced medical facilities in some regions, restrict widespread adoption.

While the integration of Artificial Intelligence (AI) holds transformative potential for revolutionizing heart failure management, it is crucial to acknowledge and critically appraise the current limitations and barriers to its widespread clinical adoption. A purely optimistic view would overlook significant challenges that require ongoing research and collaborative efforts to overcome.

One primary concern revolves around data privacy and security. AI models thrive on vast datasets, often comprising highly sensitive patient health information. Ensuring the secure collection, storage, and anonymization of this data, while adhering to stringent regulations like HIPAA and GDPR, presents a substantial hurdle. Breaches or misuse of such data could have severe ethical and legal repercussions, necessitating robust cybersecurity frameworks and clear data governance policies.

Furthermore, the interpretability of AI models, particularly complex deep learning algorithms often referred to as ‘black boxes,’ remains a significant barrier. Clinicians require transparency to trust and effectively utilize AI-driven recommendations. If a model suggests a particular therapeutic adjustment, understanding why that recommendation was made is paramount for physician confidence, accountability, and ultimately, patient safety. The current opacity of many AI decision-making processes can hinder clinician acceptance and integration into established clinical workflows.

Finally, regulatory approval and standardization for AI in medicine are still evolving. Unlike traditional medical devices, AI algorithms can adapt and learn, posing unique challenges for static regulatory frameworks. There is a pressing need for standardized protocols for AI model development, validation, and continuous monitoring to ensure reproducibility, generalizability, and long-term reliability across diverse patient populations and healthcare systems.

### 8.2. Future Directions

Several promising directions are proposed for the future of AI-guided neuromodulation in HF management to overcome these challenges and advance the field. Integrating AI-neuromodulation platforms holds significant potential, combining advanced sensors and machine learning to enable real-time monitoring and precise treatment optimization. Another key direction is the creation of personalized HF management algorithms that address the full ejection fraction spectrum, tailoring interventions to individual patient profiles, including variations in ejection fraction and comorbidities, to improve outcomes for both HFpEF and HFrEF. Additionally, hybrid approaches that combine pharmacotherapy, neuromodulation, and AI-guided care pathways offer a synergistic strategy, allowing dynamic adjustments to treatment plans based on real-time data. To support these advancements, future research should prioritize generating robust data for HFpEF populations through large-scale, multicenter trials. These trials should incorporate diverse patient cohorts, standardized outcome measures, and long-term follow-up to ensure clinical relevance and generalizability. We propose some actional steps to advance AI in HF management as below:

**Actionable Steps to Advance Artificial Intelligence in Heart Failure Neuromodulation.**

To accelerate up clinical adoption and optimize the therapeutic potential of AI-guided neuromodulation, we recommend the following priorities:

1. Conduct large-scale, phenotype-specific randomized controlled trials with AI-based patient selection to evaluate efficacy across both the HFpEF and HFrEF populations.
2. Create standardized, AI-ready multicenter datasets that combine clinical, imaging, biomarker, and device-derived data to allow for robust and generalizable model building.
3. Implement adaptive, closed-loop neuromodulation devices in clinical trials, allowing for real-time modification of stimulation parameters based on physiological feedback.
4. Ensure algorithm transparency and interpretability to increase clinician trust, facilitate regulatory assessment, and promote safe clinical integration.
5. To overcome technological, ethical, and cost-related difficulties, form interdisciplinary consortiums that include cardiologists, neurologists, biomedical engineers, data scientists, and ethicists.
6. Develop post-market surveillance systems for AI-neuromodulation devices to ensure long-term safety, performance, and equitable patient access.
7. These proposals aim to close existing evidence gaps, boost precision medicine techniques, and move AI-guided neuromodulation closer to common use in heart failure care.

## 9. Conclusions

This review highlights the critical and evolving role of artificial intelligence (AI) in revolutionizing the management of heart failure (HF), particularly through its integration with neuromodulation therapies. We have explored how AI can significantly enhance neuromodulation by facilitating precise patient selection through deep phenotyping, optimizing therapy parameters in real-time, and enabling sophisticated closed-loop systems that adapt to individual patient needs and physiological responses. The preclinical and emerging clinical evidence suggests a promising future where AI-guided neuromodulation can offer personalized and more effective interventions for both heart failure with reduced ejection fraction (HFrEF) and the heterogeneous syndrome of heart failure with preserved ejection fraction (HFpEF).

Despite the compelling potential, several significant evidence gaps and challenges remain. Current limitations include the complexity of integrating diverse data sources, ensuring the ethical oversight of AI algorithms, and navigating the path to widespread clinical adoption. Transparency and interpretability of AI models are crucial to build clinician trust and minimize biases. Furthermore, dedicated neuromodulation studies for HFpEF, especially those incorporating AI-driven adaptive approaches, are limited, necessitating more research.

Future research directions must focus on developing more robust and validated HFpEF models to rigorously test AI-guided neuromodulation strategies. Extensive, sham-controlled clinical trials are essential to unequivocally prove the long-term safety, effectiveness, and cost-effectiveness of these advanced closed-loop neuromodulation systems. Fostering interdisciplinary collaboration among cardiologists, neurologists, data scientists, and engineers will be paramount. Ultimately, continued ethical innovation and rigorous clinical validation are vital to translate the promise of AI-guided neuromodulation into personalized, data-driven, and patient-centered treatment strategies that improve outcomes for individuals living with heart failure.

## Figures and Tables

**Table 1:** Comparative Summary of Autonomic Dysregulation in HFrEF and HFpEF.

Autonomic Feature.	HFrEF Characteristics	HFpEF Characteristics
**Global Sympathetic Activity**	Markedly increased; hyperadrenergic state	Mild-moderately increased; often comorbidity-driven; less clear-cut
**Cardiac Sympathetic Activity**	Significantly elevated; drives remodeling, arrhythmias	Less consistent evidence; not universally elevated; dysfunctional signaling possible
**Parasympathetic Tone**	Markedly reduced	Variable; may be reduced, less consistently/severely; “intermediate phenotype” suggested
**Baroreflex Sensitivity (BRS)**	Significantly blunted/impaired	Often impaired; contributes to imbalance
**Chemoreflex Sensitivity**	Often hyperactive; fuels sympathetic drive, ventilatory instability	Less characterized; comorbidities (e.g., sleep apnea) may contribute
**Vascular Stiffness**	May be present; less a primary hallmark	Prominent; key to diastolic dysfunction and impaired ventricular-vascular coupling
**Chronotropic Competence**	Can be impaired; less defining than in HFpEF	Chronotropic incompetence (CI) common (30–50%); major contributor to exercise intolerance
**Key Neurohormones**	Markedly elevated	Elevated in subsets (~67% one + biomarker); elevation generally less than HFrEF
**Response to Neurohormonal Blockade**	Cornerstone therapy; significant prognostic benefit	Generally no significant prognostic benefit in broad population; potential benefit in HFmrEF

**Table 2:** Summary of Key Vagus Nerve Stimulation (VNS) Trials in HFrEF.

Trial	Design/Randomization	Sample Size/Follow-Up	Primary Endpoint(s)	Key Secondary Endpoints
ANTHEM-HF [40]	Open-label, multicenter (2:1 ON/OFF)	60 patients/6 months	LVEF, LVESV	6MWT, MLHFQ, NYHA class, LVESD, Mean Heart Rate, HRV
NECTAR-HF [41]	Randomized, sham-controlled (2:1 ON/OFF)	96 patients/6 months	Change in LVESD	6MWT, MLHFQ, NYHA class, LVESV, LVEF, NT-proBNP
INOVATE-HF [39]	Multinational, randomized (3:2 ON/OFF)	707 patients/16 months	Composite of all-cause mortality or HF hospitalization	6MWT, KCCQ, NYHA class, LVESV

**Table 3:** Major Clinical Trials of Neuromodulation for Heart Failure and Relevance to AI Integration.

Trial Acronym/Study	Neuromodulation Modality	Target Population (HF Phenotype, NYHA Class, LVEF)	AI Component (Actual or Potential/Needed)	Primary Endpoints (Examples)	Key Findings and Limitations (Re: Parameter Setting/Responder Variability)	Implications for AI-Guided Approaches
**INOVATE-HF** [39]	VNS	HFrEF, NYHA III, LVEF ≤ 40%	Manual titration; Potential for AI optimization	Composite: all-cause mortality or worsening HF	Did not meet primary endpoint. Questions re: optimal patient selection and stimulation parameters.	Highlights need for AI in patient selection, personalized parameter optimization, adaptive control.
**NECTAR-HF** [41]	VNS	HFrEF, NYHA II-III, LVEF ≤ 35%	Manual titration; Potential for AI optimization	Change in LVESD index	No significant benefit on primary/secondary endpoints. Suboptimal parameter settings suspected.	Reinforces need for AI to overcome heuristic parameter setting limitations, address inter-patient variability.
**ANTHEM-HF** [40]	VNS (right-sided)	HFrEF, NYHA II-III, LVEF ≤ 40%	Manual titration; Potential for AI optimization	LVEF, LVESV, 6MWT, MLWHFQ, NT-proBNP	Improvements in LVEF, symptoms, functional capacity. Smaller study; parameter optimization still challenging.	Suggests VNS potential; AI could enhance consistency/magnitude of benefit via optimized, adaptive therapy.
**BeAT-HF** [36,45]	BAT	HFrEF, NYHA III (or II with recent III), LVEF ≤ 35%	Physician-programmed; Potential for AI personalization and adaptation	6MWT, MLWHFQ score, NT-proBNP; MANCE rate	BAT safe; significantly improved QoL, exercise capacity, NT-proBNP. Durable QoL benefits (24mo).	Positive results form platform for AI to refine BAT (personalize, adapt).
**LINK-HF2 (Pilot)** [83]	Intervention guided by AI	HF patients	AI analytics guide interventions	Workflow, communication, clinician beliefs, notification response	Clinicians responded to AI notifications; pilot guided main trial implementation.	Demonstrates feasibility of integrating AI analytics into clinical workflows.

## Data Availability

This review was based on publicly available academic literature databases.
